# Global research on the crosstalk between intestinal microbiome and colorectal cancer: A visualization analysis

**DOI:** 10.3389/fcimb.2023.1083987

**Published:** 2023-03-15

**Authors:** Shanshan Yang, Shaodong Hao, Hui Ye, Xuezhi Zhang

**Affiliations:** ^1^ Department of Integrated Traditional Chinese and Western Medicine, Peking University First Hospital, Beijing, China; ^2^ Graduate School, Beijing University of Chinese Medicine, Beijing, China; ^3^ Spleen-Stomach Department, Fangshan Hospital, Beijing University of Chinese Medicine, Beijing, China

**Keywords:** intestinal microbiome, colorectal cancer, hotspots and trends, high-cited papers, bibliometrics

## Abstract

**Background:**

Increasing evidence has shown that the intestinal microbiome (IM) is highly linked to colorectal cancer (CRC). To investigate scientific output, identify highly cited papers, and explore research hotspots and trends in the field of IM/CRC, we conducted a bibliometric and visualized analysis.

**Methods:**

A bibliographic search regarding IM/CRC research (2012-2021) was implemented on October 17, 2022. The terms attached to IM and CRC were searched for in the titles (TI), abstracts (AB), and author keywords (AK). The main information was extracted from the Web of Science Core Collection (WoSCC). Biblioshiny from R packages and VOSviewer were used for data visualization.

**Results:**

A total of 1725 papers related to IM/CRC were retrieved. Publications on IM/CRC have grown rapidly from 2012 to 2021. China and the United States were in the leading position for publications in this field and made the most significant contributions to IM/CRC research. Shanghai Jiao Tong University and Harvard University were the most productive institutions. The high-yield authors were Yu Jun and Fang Jing Yuan. The International Journal of Molecular Sciences published the most papers, whereas Gut had the most citations. Historical citation analysis showed the evolution of IM/CRC research. Current status and hotspots were highlighted using keyword cluster analysis. The hot topics include the effect of IM on tumorigenesis, the effect of IM on CRC treatment, the role of IM in CRC screening, the mechanisms of IM involvement in CRC, and IM modulation for CRC management. Some topics, such as chemotherapy, immunotherapy, *Fusobacterium nucleatum* and short-chain fatty acids could be the focus of IM/CRC research in the coming years.

**Conclusion:**

This research evaluated the global scientific output of IM/CRC research and its quantitative features, identified some significant papers, and gathered information on the status and trends of IM/CRC research, which may shape future paths for academics and practitioners.

## 1 Introduction

Colorectal cancer (CRC) is the third most common cancer and second leading cause of cancer-related deaths worldwide (Sung et al., 2021), seriously endangering human health. Early screening and intervention are crucial for patients with CRC. In recent years, the intestinal microbiome (IM) has emerged as a pathogenic factor in many diseases. The link between GM and CRC has become a hot topic, and IM plays an essential regulatory role in the incidence, development, and treatment of cancer (Janney et al., 2020; Kim and Lee, 2021; Ma et al., 2022). Multiple studies have shown that IM can alter the susceptibility and progression of CRC by modulating inflammatory responses and immune function, mediating DNA damage and repair, and generating metabolites involved in cancer progression or downregulation (Sánchez-Alcoholado et al., 2020; Xing et al., 2022). Moreover, IM can influence and modify the antitumor efficacy and toxicity of cancer treatment, which can impede the potential clinical use of antitumor drugs. IM can also be used as a biomarker to predict cancer patients’ prognosis. IM changes may directly contribute to carcinogenesis and progression, and modulation of IM may be an approach for preventing and treating CRC (Louis et al., 2014; Gagnière et al., 2016; O'Keefe, 2016; Tilg et al., 2018; Janney et al., 2020).

Bibliometrics is an interdisciplinary science that quantifies all knowledge bearers utilizing mathematics and statistics, which realizes the visualization of hot topics and knowledge evolution in specific research fields, and has been used successfully in medical studies. Many cancer-related issues have been thoroughly investigated using bibliometrics, such as high-cited papers in cancer immunotherapy (Liu et al., 2022), immunotherapy for hepatocellular carcinoma (Shen et al., 2022) and CRC (Ma et al., 2022), links between IM and cancer (Zyoud et al., 2022), and links between IM and cancer immunotherapy (Yang S. et al., 2022). In the past decade, clinical and animal studies on the link between IM and CRC have steadily increased. However, there is currently no research on the quantitative investigation of the link between IM and CRC. This paper attempts to identify IM/CRC-related research in the last decade and analyze its features, systematically review the crosstalk between IM and CRC, and conduct visualized analysis and knowledge mapping of bibliometric indicators such as research hotspots, hot topics, and publishing institutions in the field of IM/CRC, and seeks to help scholars better grasp the dynamic changes and development trends of IM/CRC-related research.

## 2 Materials and methods

### 2.1 Data sources and search methods

The WoSCC is an important database for obtaining global academic resources, including various academic journals. With a stringent screening process based on Bradford’s law, the WoSCC’s Science Citation Index Expanded (SCI-E) highlights the most reputable and noteworthy academic work in natural science (Yang S. et al., 2022; Zyoud et al., 2022). Therefore, it was selected as the data source.

**Figure 1:**
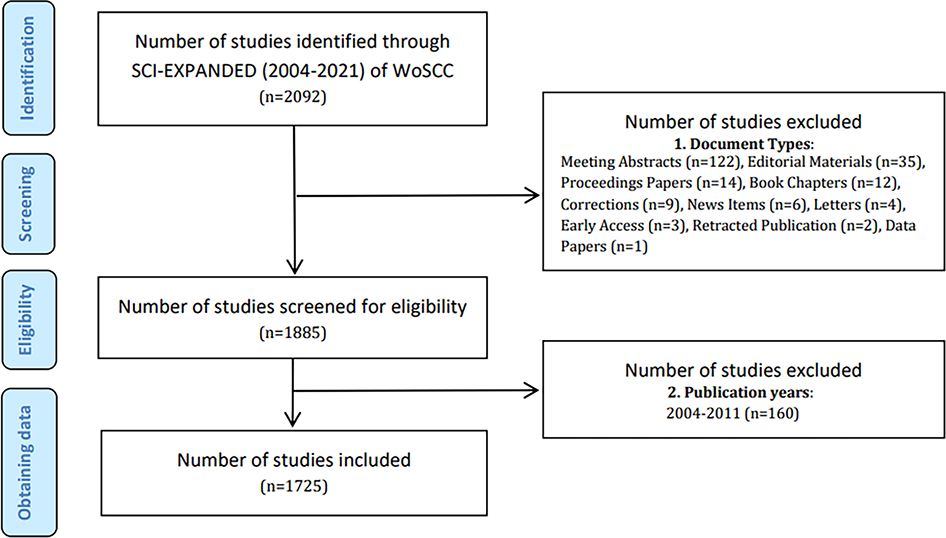
Flow chart of literature screening in IM/CRC.

All search results were performed and retrieved from the SCI-E of the WoSCC database on October 17, 2022. The search used the following terms: TI OR AB OR AK = “colorectal neoplasm” and “intestinal microbiome” and their synonyms based on the “advanced search” method. Synonyms related to colorectal neoplasm and intestinal microbiome were obtained from the Medical Subject Headings (MeSH) in PubMed. The search strategy is presented in **Supplementary Material S1**. The screening standards comprised: (1) publication date from 2021-01-01 to 2021-12-31; (2) the literary categories adopted “article” and “review”. Finally, 1725 papers were acquired (**Figure 1**), with 1187 records for “articles” and 538 records for “reviews”. Two researchers (SY and SH) independently performed the search and data extraction. We refined the essential information from the raw data and saved it in text format.

### 2.2 Data analysis and software applications

Scientometric analysis was performed using Biblioshiny of Bibliometrix in the R-package (version 4.1.3, Boston, MA, USA), VOSviewer (version 1.6.18, Leiden University, the Netherlands), a data visualization website (https://www.bioinformatics.com.cn/), and Microsoft Excel 2019 (Microsoft, Redmond, Washington, USA). Bibliometrix provides a set of scientometric analytical tools. VOSviewer was used to create and visualize bibliometric networks. Specifically, machine learning was used to evaluate the distribution of each component, including annual scientific production, most relevant journals, authors, affiliations, or countries; most local impact journals, authors, affiliations, or countries by H-index or total citation (TC); annual production of top journals or authors or affiliations or countries over time; main financial agencies; country scientific output; country collaboration network; historical direct citation network; high-cited papers; high-impact factor (IF) papers; common keywords; and cluster analysis. The “2021 Incites Journal Citation Report” defined the journal’s JCR Quartile and IF.

## 3 Results

### 3.1 Annual scientific output

**Figure 2:**
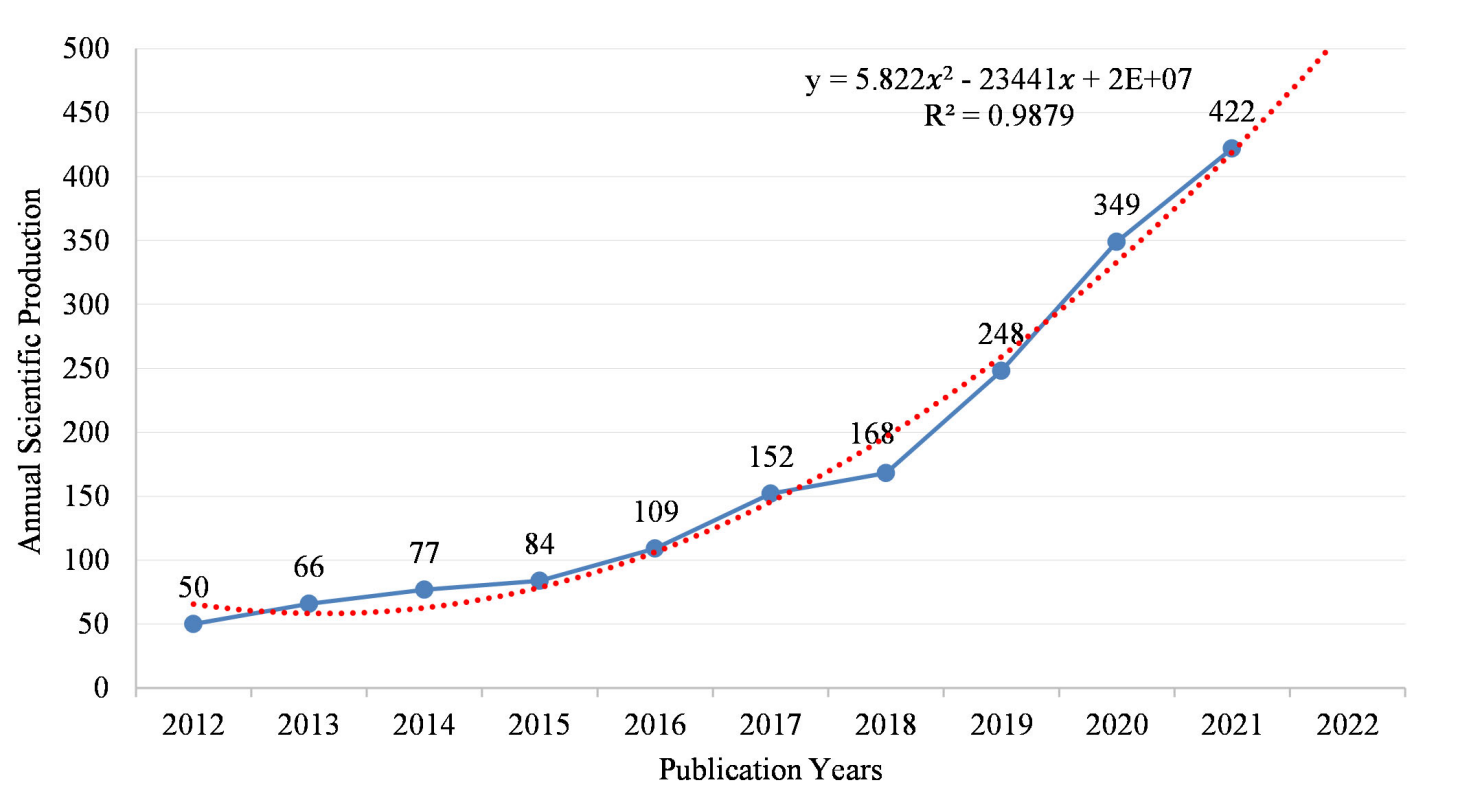
Annual scientific output and the polynomial fitting curve of output in IM/CRC.

**Figure 2** shows the annual number of papers (Np) in IM/CRC research from 2012 to 2021, in which the annual Np showed an increasing trend (annual growth rate, 37.20%). From 2012 to 2018, the Np increased slowly (annual growth rate, 16.86%), which demonstrated that IM/CRC research was in a stage of stable development. Since 2019, the Np has increased rapidly (annual growth rate, 58.00%), showing that IM/CRC research entered a period of rapid development. To predict Np in 2022, we used a polynomial regression model: (*f(x)=p_0_x^n^+p_1_x^n-1^+p_2_x^n−2^+p_3_x^n−3^+…+p_n_*). By fitting the data of the annual output, a fitting curve model was created, with the formula y=5.822 *x* ^2^-23441 *x*+2E+07, and the fitting degree (R² = 0.9879) was excellent. Based on this formula, the Np in IM/CRC is estimated to reach approximately 500 in 2022.

### 3.2 Main journals

**Figure 3:**
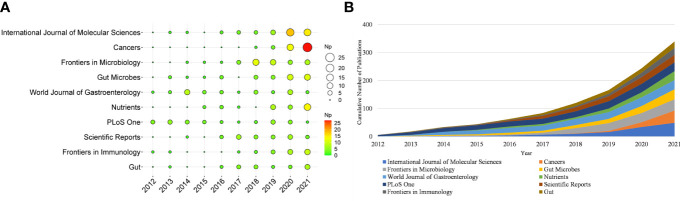
**(A)** Annual scientific production of the top 10 productive journals over time in IM/CRC (the size of the circle represents the number of papers, and the larger the circle, the more output). **(B)** Cumulative scientific output of the top 10 productive journals in IM/CRC.

More than 500 journals participated in this writing process. **Table 1** shows the top ten productive journals, of which *International Journal of Molecular Sciences* was the most productive (n = 49), followed by *Cancers* (n = 44), and *Frontiers in Microbiology* (n = 40). **Figure 3A** depicts the top ten journals’ annual NP, with *Cancers* becoming the most productive journal in 2021. **Figure 3B** summarizes the cumulative output of the top ten journals. The Np in these journals was 340, accounting for approximately 19.71% of the total output, indicating their excellent production capacity for IM/CRC research. TC can show periodic significance, and the H-Index can assess the periodical academic impact. **Table 2** shows the top ten highly cited journals, of which *Gut* ranked first, followed by *World Journal of Gastroenterology*, *PLoS One*, *Science* and *Nature Reviews Gastroenterology & Hepatology*. In the H-index, *World Journal of Gastroenterology* (n = 23) ranked first, followed by *Gut* (n = 20), and *Frontiers in Microbiology* (n = 20).

**Table 1:** The top 10 productive journals in IM/CRC.

Rank	Journals	Np	TC	H-index	IF	JCR	Countries
1	International Journal of Molecular Sciences	49	1728	19	6.208	Q1	Switzerland
2	Cancers	44	584	13	6.575	Q1	Switzerland
3	Frontiers in Microbiology	40	1679	20	6.064	Q1	Switzerland
4	Gut Microbes	36	1101	16	9.434	Q1	USA
5	World Journal of Gastroenterology	33	2418	23	5.374	Q2	USA
6	Nutrients	32	923	14	6.706	Q1	Switzerland
7	PLoS One	31	2131	19	3.752	Q2	USA
8	Scientific Reports	27	863	18	4.996	Q2	UK
9	Frontiers in Immunology	25	1050	15	8.786	Q1	Switzerland
10	Gut	23	4149	20	31.793	Q1	UK

**Table 2:** The top 10 local impact journals in IM/CRC.

Rank	Journals	TC	Journals	H-index
1	Gut	4149	World Journal of Gastroenterology	23
2	World Journal of Gastroenterology	2418	Gut	20
3	PLoS One	2131	Frontiers in Microbiology	20
4	Science	1934	PloS One	19
5	Nature Reviews Gastroenterology & Hepatology	1914	Gastroenterology	19
6	Nature Reviews Microbiology	1845	International Journal of Molecular Sciences	19
7	Nature Medicine	1836	Scientific Reports	18
8	Gastroenterology	1772	Gut Microbes	16
9	Cell	1739	Frontiers in Immunology	15
10	International Journal of Molecular Sciences	1728	Nutrients	14

### 3.3 Main authors

**Figure 4:**
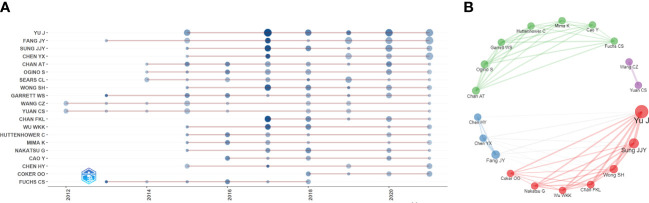
**(A)** Annual output of the top 20 productive authors over time in IM/CRC (sizes of the circle signify scientific output, and the larger the circle, the more scientific output; color depth of the circle indicates the annual citations, and the darker the color, the more citations). **(B)** Co-authorship network of the top 20 productive authors (remove isolated nodes) in IM/CRC (each node represents an author, and its size represents scientific output, each color represents a cooperative group, each line represents a coordination relation and its thickness represents cooperation intensity).

The papers included more than 9000 authors. Given the name abbreviation-caused repetition, we used the full name for the analysis. **Table 3** lists the top ten productive authors (including their TC and H-index), of which Yu Jun, Fang Jing-Yuan, Sung Joseph JY, Chen Ying-Xuan, and Ogino Shuji ranked in the top five. Yu Jun had the largest Np and H-index, and Garrett Wendy S had the highest TC, showing that their papers were of high quality and had a significant impact on IM/CRC research. **Figure 4A** shows the annual output of the top 20 authors. We found that their most influential papers appeared in 2017, and they had at least one paper in 2021. **Figure 4B** depicts the collaborative relations of the top 20 authors, of which Yu Jun, Sung Joseph JY, Wong Sunny H, and Chan Francis KL from the *Chinese University of Hong Kong* had the closest cooperative relationship, which can be called a cooperative group. Other academic groups included Fang Jing-Yuan, Chen Ying-Xuan, and Chen Hao-Yan from *Shanghai Jiao Tong University*; Chan Andrew T, Ogino Shuji, Garrett Wendy S, and Huttenhower Curtis from *Harvard Medical School*; and Wang Chong-Zhi and Yuan Chun-Su from *the University of Chicago*.

**Table 3:** The top 10 productive authors in IM/CRC.

Rank	Authors	Np	TC	H-index	Affiliations	Countries
1	Yu, Jun	29	2929	21	Chinese Univ Hong Kong	China
2	Fang, Jing-Yuan	25	1607	11	Shanghai Jiao Tong Univ	China
3	Sung, Joseph J. Y.	17	2074	15	Chinese Univ Hong Kong	China
4	Chen, Ying-Xuan	16	1219	8	Shanghai Jiao Tong Univ	China
5	Ogino, Shuji	15	1570	11	Harvard Med Sch	USA
6	Chan, Andrew T.	15	2060	14	Harvard Med Sch	USA
7	Wong, Sunny Hei	14	2072	12	Chinese Univ Hong Kong	China
8	Sears, Cynthia L.	14	1277	12	Johns Hopkins Univ	USA
9	Garrett, Wendy S.	13	3081	12	Harvard Med Sch	USA
10	Yuan, Chun-Su	13	470	10	Univ Chicago	USA

### 3.4 Major countries/regions and institutions

**Figure 5:**
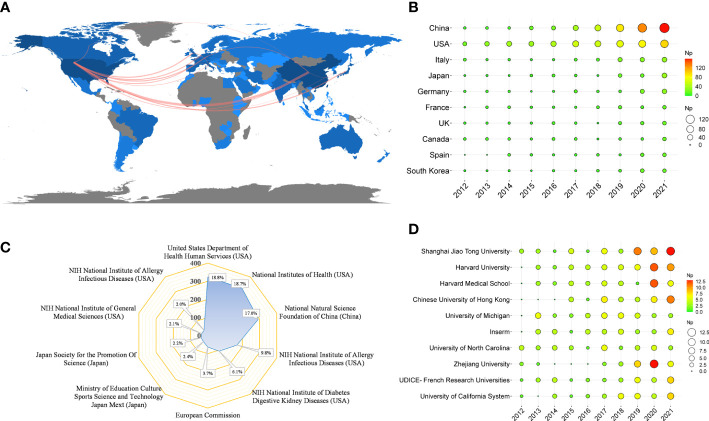
**(A)** Country scientific production and international collaboration network (min edges set to 10) in IM/CRC. **(B)** Annual output of the top 10 productive countries over time in IM/CRC. **(C)** The top 10 funding agencies and source countries in IM/CRC research. **(D)** Annual output of the top 10 institutions over time in IM/CRC.

**Table 4** shows that the papers were mainly from China (n = 527) and the United States (n = 524), accounting for about 61% of the total output. **Figure 5A** depicts the country’s scientific production and the main national collaboration network. Among them, the United States was a leader in international cooperation and had the closest relationship with China. **Figure 5B** depicts the annual Np of the top ten countries. The United States held the top spot in annual Np until 2019, when China overtook the United States.

**Table 4:** The top 10 productive countries and institutions in IM/CRC.

Rank	Countries	Np	TC	H-index	Institutions	Np	TC	H-index
1	China	527	19917	65	Shanghai Jiao Tong University	55	4450	26
2	USA	524	36168	94	Harvard University	51	5186	28
3	Italy	120	6063	38	Harvard Medical School	40	4797	26
4	Japan	91	5356	27	Chinese University of Hong Kong	38	3050	23
5	Germany	87	5026	30	University of Michigan	37	5479	27
6	France	71	5323	34	Inserm	35	3228	21
7	UK	68	5235	29	Zhejiang University	35	1707	20
8	Canada	67	6135	31	University of North Carolina	35	4352	21
9	Spain	59	3072	28	UDICE-French Research Universities	34	2040	19
10	South Korea	58	1494	24	University of California System	34	1790	18

**Table 4** also shows the top ten most productive institutions, of which *Shanghai Jiao Tong University*, *Harvard University*, *Harvard Medical School*, *Chinese University of Hong Kong*, and *the University of Michigan* were among the top five. **Figure 5C** illustrates the main financial agencies involved. These agencies were mainly from the United States, China, and Japan, indicating strong support for IM/CRC-related research. **Figure 5D** shows the annual Np of the top ten institutions between 2012 and 2021. Among these, *Shanghai Jiao Tong University* began earlier and published the most papers.

### 3.5 Analysis of cited papers in IM/CRC research

#### 3.5.1 Top 20 most cited articles in IM/CRC research

High-cited articles are one of the most valuable indicators in bibliometrics with extremely high academic importance in a field. **Table 5** lists the top 20 high-cited papers in original research (published between 2012 and 2020). Based on the subject matter, we summarized the following three points:

**Table 5:** The top 20 most cited original research in IM/CRC.

Rank	Title	First author	Year	Journals	IF	JCR	TC
1	Intestinal Inflammation Targets Cancer-Inducing Activity of the Microbiota	Arthur, JC	2012	Science	63.714	Q1	1280
2	Fusobacterium nucleatum Potentiates Intestinal Tumorigenesis and Modulates the Tumor-Immune Microenvironment	Kostic, AD	2013	Cell Host Microbe	31.316	Q1	1233
3	Activation of Gpr109a, Receptor for Niacin and the Commensal Metabolite Butyrate, Suppresses Colonic Inflammation and Carcinogenesis	Singh, N	2014	Immunity	43.474	Q1	1126
4	Fusobacterium nucleatum infection is prevalent in human colorectal carcinoma	Castellarin, M	2012	Genome Res.	9.438	Q1	1074
5	Fusobacterium nucleatum Promotes Chemoresistance to Colorectal Cancer by Modulating Autophagy	Yu, TC	2017	Cell	66.850	Q1	783
6	Structural segregation of gut microbiota between colorectal cancer patients and healthy volunteers	Wang, TT	2012	ISME J.	11.217	Q1	712
7	Gut microbiome development along the colorectal adenoma-carcinoma sequence	Feng, Q	2015	Nat. Commun.	17.694	Q1	603
8	Potential of fecal microbiota for early-stage detection of colorectal cancer	Zeller, G	2014	Mol. Syst. Biol.	13.068	Q1	543
9	Human Gut Microbiome and Risk for Colorectal Cancer	Ahn, J	2013	JNCI-J. Natl. Cancer Inst.	11.816	Q1	532
10	Metagenomic analysis of faecal microbiome as a tool towards targeted non-invasive biomarkers for colorectal cancer	Yu, J	2017	Gut	31.793	Q1	460
11	Two FOXP3(+)CD4(+) T cell subpopulations distinctly control the prognosis of colorectal cancers	Saito, T	2016	Nat. Med.	87.241	Q1	446
12	Patients with familial adenomatous polyposis harbor colonic biofilms containing tumorigenic bacteria	Dejea, CM	2018	Science	63.714	Q1	435
13	Fusobacterium nucleatum in colorectal carcinoma tissue and patient prognosis	Mima, K	2016	Gut	31.793	Q1	420
14	The Gut Microbiome Modulates Colon Tumorigenesis	Zackular, JP	2013	mBio	7.786	Q1	412
15	Human Intestinal Lumen and Mucosa-Associated Microbiota in Patients with Colorectal Cancer	Chen, WG	2012	PLoS One	3.752	Q2	398
16	Stool Microbiome and Metabolome Differences between Colorectal Cancer Patients and Healthy Adults	Weir, TL	2013	PLoS One	3.752	Q2	389
17	NOD2-mediated dysbiosis predisposes mice to transmissible colitis and colorectal cancer	Couturier-Maillard, A	2013	J. Clin. Invest.	19.456	Q1	360
18	Wild Mouse Gut Microbiota Promotes Host Fitness and Improves Disease Resistance	Rosshart, SP	2017	Cell	66.850	Q1	357
19	Metagenomic and metabolomic analyses reveal distinct stage-specific phenotypes of the gut microbiota in colorectal cancer	Yachida, S	2019	Nat. Med.	87.241	Q1	346
20	Tumour-associated and non-tumour-associated microbiota in colorectal cancer	Flemer, B	2017	Gut	31.793	Q1	340

First, there are differences in IM between patients with CRC and healthy controls (Chen et al., 2012; Wang T. et al., 2012; Ahn et al., 2013; Weir et al., 2013). Microbiome transformation may occur in the early stages of CRC and fecal metagenomes may reveal microbial characteristics specific to CRC (Yachida et al., 2019). Tumor and non-tumor related microbiota in CRC may be different; mucosal microbiota is only partially reflected in fecal microbiota, and CRC can be stratified based on the higher-level mucosal microbiota co-abundance group (Flemer et al., 2017). Furthermore, IM can be used for CRC screening. Metagenomic analysis of GM can be used as a tool for targeting noninvasive biomarkers to diagnose CRC (Yu J. et al., 2017). The sensitivity of CRC diagnosis can be increased by combining IM with the standard fecal occult blood test (FOBT) (Zeller et al., 2014). Second, IM may play a key role in tumorigenesis, inflammation-cancer, and adenoma-carcinoma transition. for example, laboratory-type mice reconstructed with IM from wild-type mice exhibited improved resistance to colorectal tumorigenesis (Rosshart et al., 2017). Moreover, germ-free mice colonized with IM from tumor-bearing mice had a relatively higher population abundance related to inflammation-driven tumor formation (Zackular et al., 2013). NOD_2_-mediated ecological imbalance made mice susceptible to colitis and CRC (Couturier-Maillard et al., 2013), whereas activating Gpr109a (the receptor of the symbiotic metabolite butyrate) inhibited colitis and carcinogenesis (Singh et al., 2014). Moreover, inflammation can promote CRC, and IM has been identified as an inflammatory target that affects CRC progression (Arthur et al., 2012). IM can also evolve along the colorectal adenoma-carcinoma sequence (Feng et al., 2015). Patients with familial adenomatous polyposis carry colonic biofilms containing carcinogenic bacteria, and tumor-prone mice colonized with carcinogenic bacteria show a faster tumor onset and higher mortality (Dejea et al., 2018). Third, *Fusobacterium nucleatum* (*F. nucleatum*) is not only a key pathogenic factor, but also a biomarker for the therapeutic effect of CRC. *F. nucleatum* infection is prevalent in human CRC (Castellarin et al., 2012) and may potentiate intestinal tumorigenesis, regulate the tumor microenvironment (Kostic et al., 2013), and promote chemoresistance to CRC by regulating autophagy (Yu T. et al., 2017). Moreover, the amounts of *F. nucleatum* and the IM-produced cytokines IL-12 and TGF-β have been shown to be different, which led to different proportions of lymphocytes in different CRCs (Saito et al., 2016). The amount of *F. nucleatum* in CRC tissues is also related to shorter survival and may act as a prognostic biomarker (Mima et al., 2016).

#### 3.5.2 Top 10 most cited reviews in IM/CRC research

**Table 6** shows the top ten high-cited reviews (issued between 2012 and 2019), nearly half were from *Nature Reviews Gastroenterology & Hepatology* (n = 2) and *Nature Reviews Microbiology* (n = 2). Two review articles (Marchesi et al., 2016; Kho and Lal, 2018) outlined the key role of IM in host health and disease. Several review articles (Louis et al., 2014; Gagnière et al., 2016; O'Keefe, 2016; Tilg et al., 2018) outlined the links between diet, IM and metabolites, and CRC. Furthermore, some reviews detailed the mechanisms of inflammation-driven IM dysbiosis (Zeng et al., 2017), outlined the interplay of bile acids and microbiota in gastroenteritis inflammation and carcinogenesis (Jia et al., 2018), mentioned the theoretical hypothesis of the “driver-passenger” model, arguing that some microbes may cause adenomas and cancers (Tjalsma et al., 2012), and summarized the impact of IM on tryptophan metabolism-mediated intestinal immunity (Gao et al., 2018).

**Table 6:** The top 10 most cited reviews in IM/CRC.

Rank	Title	First author	Year	Journals	IF	JCR	TC
1	The gut microbiota, bacterial metabolites and colorectal cancer	Louis, P	2014	Nat. Rev. Microbiol.	78.297	Q1	1372
2	The gut microbiota and host health: a new clinical frontier	Marchesi, JR	2016	Gut	31.793	Q1	1183
3	Bile acid-microbiota crosstalk in gastrointestinal inflammation and carcinogenesis	Jia, W	2018	Nat. Rev. Gastroenterol. Hepatol.	73.082	Q1	551
4	Diet, microorganisms and their metabolites, and colon cancer	O’Keefe, SJD	2016	Nat. Rev. Gastroenterol. Hepatol.	73.082	Q1	479
5	Impact of the Gut Microbiota on Intestinal Immunity Mediated by Tryptophan Metabolism	Gao, J	2018	Front. Cell. Infect. Microbiol.	6.073	Q2	477
6	A bacterial driver-passenger model for colorectal cancer: beyond the usual suspects	Tjalsma, H	2012	Nat. Rev. Microbiol.	78.297	Q1	473
7	Gut microbiota imbalance and colorectal cancer	Gagniere, J	2016	World J. Gastroenterol.	5.374	Q2	385
8	The Human Gut Microbiome - A Potential Controller of Wellness and Disease	Kho, ZY	2018	Front. Microbiol.	6.064	Q1	367
9	The Intestinal Microbiota in Colorectal Cancer	Tilg, H	2018	Cancer Cell	38.585	Q1	320
10	Mechanisms of inflammation-driven bacterial dysbiosis in the gut	Zeng, MY	2017	Mucosal Immunol.	8.701	Q1	315

#### 3.5.3 Top 20 most cited references in IM/CRC research

**Figure 6:**
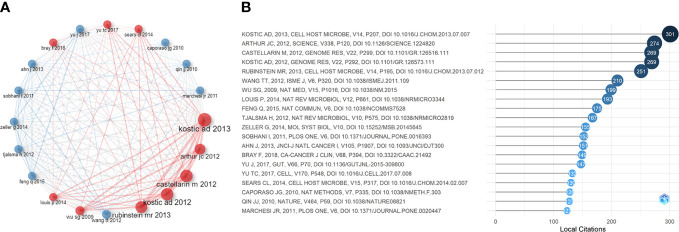
**(A)** Co-citation network of the top 20 most cited references in IM/CRC research. **(B)** The top 20 most cited references in IM/CRC research.

Considering that some classical papers (particularly before 2012) still had important significance, we searched for the most cited references to find important papers that may have been ignored. **Figure 6A** and **Figure 6B** show the top 20 most-cited references and their citation relationships. In 2009, Wu et al. (2009) demonstrated that human colon bacteria could promote colon tumorigenesis by activating the T-assisted type 17 T cell response. In 2010, Caporaso et al. (2010) developed QIIME (a tool for unpacking massive high-throughput sequencing data). A human intestinal metagenomic study (Qin et al., 2010) based on metagenomic sequencing has provided a broad perspective on the important functions of intestinal bacteria. These technologies have increased support for IM/CRC research. In 2011, Sobhani et al. (2011) applied pyrosequencing technology to report that colon cancer is associated with microbial dysbiosis, opening up a new field for CRC screening and pathophysiology research. Marchesi et al. (2011) compared the microbial composition between colon tumors and the adjacent non-malignant colonic mucosa, revealing significant differences in the IM of the two sites.

#### 3.5.4 Historical cited papers in IM/CRC research

**Figure 7:**
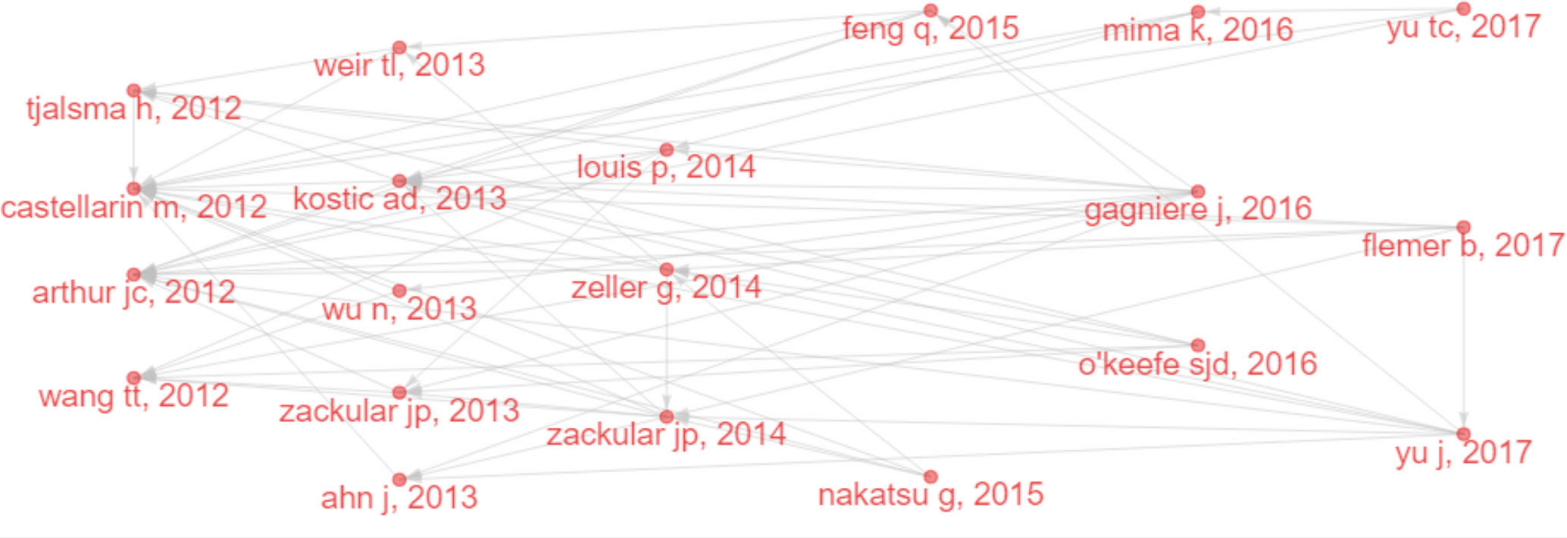
Historical direct citation network in IM/CRC (gray lines indicate the citation relations, and each dot represents a paper by author and year).

Several classic papers were identified through historiographic analysis (**Figure 7**). Some papers had been mentioned in the part of most-cited articles and reviews. Moreover, Wu et al. (2013) showed that the IM of CRC was characterized by the enrichment of potential pathogens, such as *Fusobacterium* and *Campylobacter* and the reduction of butyrate-producing bacteria. Zackular et al. (2014) showed that IM can be used as a screening tool to detect precancerous lesions and cancers in CRC. Nakatsu et al. (2015) classified IM communities in the intestinal mucosa at different stages of CRC and found that with the progression of CRC along the “adenoma-cancer” sequence, mucosal microflora can establish a microecosystem. In 2014, a review (Louis et al., 2014) outlined the links between diet, metabolism, and CRC, showing that short-chain fatty acids such as acetates, propionates, and butyrate can inhibit inflammation and cancer, while some microbial metabolites (such as secondary bile acids) can promote carcinogenesis. In 2016, a review (O'Keefe, 2016) summarized the links between diet, microorganisms and their metabolites, and colon cancer, showing that meat increases the risk of colon cancer, but foods rich in fiber inhibit the risk, which may be related to IM. A review (Gagnière et al., 2016) discussed the relationship between IM and CRC, with an emphasis on dysbacteriosis and potential characteristics of carcinogenic bacteria, such as genotoxicity and other virulence factors, inflammation, host defense regulation, bacterial metabolism, oxidative stress, and antioxidant defense regulation.

### 3.6 Analysis of high-IF papers in IM/CRC research

IF is an international universal evaluation index used to assess the influence of journals and academic quality of papers. Given that highly cited papers were mainly issued in high-IF journals, we looked for IM/CRC-related papers in high-IF journals (IF > 40) (**Supplementary Material S2**).

Twenty original articles were extracted. Among them, *Nature Medicine* (n = 5) and *Cell* (n = 5) had the most publications, followed by *Immunity* (n = 3) and *Science* (n = 3). the correlation research between CRC and IM (Rosshart et al., 2017; Dejea et al., 2018) such as *F. nucleatum* (Yu T. et al., 2017; Serna et al., 2020), genotoxic *Escherichia coli* (Pleguezuelos-Manzano et al., 2020) and *Streptococcus gallolyticus* (Boleij and Tjalsma, 2013), the mechanism research of IM/CRC from gene expression (Man et al., 2015; Wilson et al., 2019), metabolism (Belcheva et al., 2014; Singh et al., 2014; Hu et al., 2015; Scott et al., 2017), inflammation (Arthur et al., 2012) and immunity (Rosshart et al., 2017; Roberti et al., 2020; Overacre-Delgoffe et al., 2021), the intestinal fungi research (Malik et al., 2018), and the metagenomics research (Thomas et al., 2019; Wirbel et al., 2019; Yachida et al., 2019) that find characteristic IM for CRC screening has become the research focus of IM/CRC in high-IF journals over the past decade.

Among the review articles, the top ten high-IF reviews were published between 2016 and 2019, which were mainly from *Nature Reviews Gastroenterology & Hepatology* (n = 6) and *Nature Reviews Microbiology* (n = 2). Apart from the above four high-cited reviews (Tjalsma et al., 2012; Louis et al., 2014; O'Keefe, 2016; Jia et al., 2018), there were six high-IF reviews worthy of attention. Several review articles (Carbonero et al., 2012; Cani and Jordan, 2018; Janney et al., 2020) further described that the relationship between IM-mediated inflammation, metabolites, colonic gases, and CRC. One review (Wong and Yu, 2019) elaborated on the mechanism of interaction between IM and CRC, and the prospect of modulating IM for CRC management. Two reviews (Hofseth et al., 2020; Akimoto et al., 2021) discussed the key role of IM in early-onset CRC.

### 3.7 Analysis of keywords in IM/CRC research

#### 3.7.1 Analysis of high-frequency keywords

To identify the hot topics and central issues in IM/CRC research, it is necessary to examine key index-keywords (Yang S. et al., 2022; Zyoud et al., 2022). In this research, a total of 6851 keywords included 3144 author keywords and 3707 keywords plus were acquired from publications.

Highly frequent author keywords (remove main search terms) included “inflammation”, “probiotics”, “inflammatory bowel disease”, “diet”, “butyrate”, “*Fusobacterium nucleatum*”, “chemotherapy”, “ulcerative colitis”, “apoptosis”, “ulcerative colitis”, “prebiotics”, “short-chain fatty acids”, “carcinogenesis”, “metagenomics”, “biomarkers”, “bile acids”. Highly frequent keywords plus (remove main search terms) included “inflammation”, “*Fusobacterium-nucleatum*”,”chain fatty-acids”, “inflammatory-bowel-disease”, “ulcerative-colitis”, “carcinogenesis”, “tumorigenesis”, “diet”, “*Escherichia-coli*”, “butyrate”, “NF-kappa-B”, “Crohn’s-disease”, “dietary fiber”, “metabolism”, “probiotics”, “metabolites”, “oxidative stress”, “enterotoxigenic *Bacteroides-fragilis*”.

#### 3.7.2 Cluster analysis of high-frequency keywords

**Figure 8:**
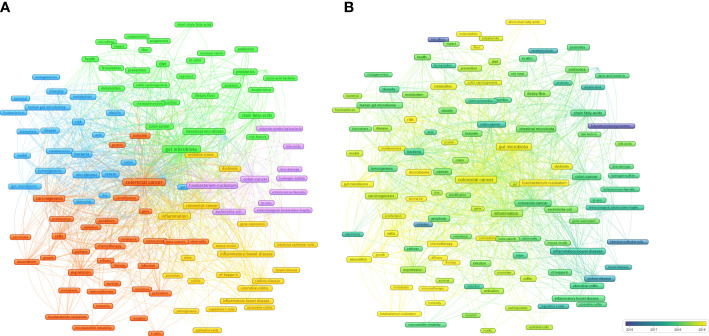
**(A)** Cluster analysis of common keywords in IM/CRC (different colors signify different clusters, and sizes of the circle signify the frequency of keyword occurrence). **(B)** Evolution trends of common keywords over time in IM/CRC (blue boxes signify the early keywords and yellow boxes signify the late keywords).

A cluster analysis of high-frequency keywords can identify hot topics in a field (Yang S. et al., 2022; Zyoud et al., 2022). A cluster analysis was conducted based on the co-occurrence of common keywords (frequency ≥ 20). Each clustered keyword unit was considered a category based on the same color (**Figure 8A**).

##### 3.7.2.1 Cluster 1

(blue nodes) focused on the links between IM, tumorigenesis, and CRC screening, such as IM as a biomarker to predict tumorigenesis risk and screen for CRC through metagenomics and metabolomics analysis, and the role of IM metabolites in CRC.

##### 3.7.2.2 Cluster 2

(red nodes) focused on the association between IM, CRC treatment (such as chemotherapy and immunotherapy) and CRC prognosis (such as metastasis, efficacy and survival).

##### 3.7.2.3 Cluster 3

(yellow nodes) focused on the mechanisms by which IM affects CRC, especially colitis-associated CRC (inflammatory bowel disease, ulcerative colitis), including inflammation (NF-kappa-B), immunity (such as intestinal epithelial cells and regulatory T cells), gene-expression and oxidative stress.

##### 3.7.2.4 Cluster 4

(purple nodes) was related to specific IM and their metabolites in CRC, including butyrate-producing bacteria, *Fusobacterium-nucleatum*, *Escherichia-coli*, *Enterococcus-faecalis*, enterotoxigenic *Bacteroides-fragilis*, bile-acids and hydrogen-sulfide.

##### 3.7.2.5 Cluster 5

(green nodes) focused on the important roles of diet (such as diet, red meat, nutrition and dietary fiber), metabolites (short-chain fatty acids), probiotics (*Lactobacillus*) and prebiotics in CRC.

#### 3.7.3 Trend analysis of high-frequency keywords

Evolving keywords can reflect frontier knowledge in a special field. We predicted the trends in IM/CRC research in the next few years using overlay visualization in VOSviewer. As shown in **Figure 8B**, more yellow nodes were found in clusters 1, 2, and 5 than that in the other clusters, and the main keywords included search terms such as “colorectal cancer”, “gut microbiota” and “gut microbiome”, and other terms such as “chemotherapy”, “immunotherapy”, “therapy”, “efficacy”, “biomarker”, “*Fusobacterium nucleatum*”, “metastasis”, “metabolites”, “short-chain fatty acids”, “immunity”.

## 4 Discussion

IM plays a key role in tumorigenesis, tumor screening, and cancer treatment (Yang S. et al., 2022; Zyoud et al., 2022), and the links between IM and CRC have received considerable attention from scholars, clinicians, and journals (Louis et al., 2014; Gagnière et al., 2016; O'Keefe, 2016; Tilg et al., 2018). In the past decade, as knowledge of IM deepens (Marchesi et al., 2016; Kho and Lal, 2018), increasing research suggests IM can affect the onset and progression of CRC and alter the efficacy and toxicity of tumor treatment (Wong and Yu, 2019; Fong et al., 2020; Sánchez-Alcoholado et al., 2020; Kim and Lee, 2021), studies on the links between IM and CRC have gradually increased, resulting in numerous research achievements. Therefore, this study carried out a bibliometric analysis of IM/CRC research, which provided researchers with a basic idea of the current status and trends in the crosstalk between IM and CRC.

### 4.1 Analysis of document issuance in IM/CRC

From the view of annual Np, a steady growth stage occurred during 2012-2018, while a rapid growth stage occurred during 2019-2021. In 2010, an important high-throughput sequencing tool-QIIME (Caporaso et al., 2010) and a human IM gene catalog generated by metagenomic sequencing, identified new research directions for IM studies (Qin et al., 2010). Henceforth, IM and CRC gradually began to collide, and an increasing number of countries began to develop microbiome projects, which promoted rapid developments in IM research. Correspondingly, IM/CRC research has begun to increase gradually. In 2019, three blockbuster metagenomic studies (Thomas et al., 2019; Wirbel et al., 2019; Yachida et al., 2019) in *Nature Medicine* emphasized the importance of IM as a potential biomarker and constructed accurate disease predictive models. Since then, IM/CRC research has received increasing attention from researchers.

Our study showed that the *International Journal of Molecular Sciences*, *Cancers* and *Frontiers in Microbiology* ranked among the top three in Np, *World Journal of Gastroenterology* had the highest H-index, and *Gut* had the highest TC. High-level academic journals easily attract the attention of scholars. The top 20 highly cited articles and high-IF articles were mainly published in *Nature Medicine* (Saito et al., 2016; Thomas et al., 2019; Wirbel et al., 2019; Yachida et al., 2019; Roberti et al., 2020), *Cell* (Belcheva et al., 2014; Man et al., 2015; Rosshart et al., 2017; Scott et al., 2017; Yu T. et al., 2017), followed by *Science* (Arthur et al., 2012; Dejea et al., 2018; Wilson et al., 2019), *Gut* (Mima et al., 2016; Flemer et al., 2017; Yu J. et al., 2017), and *Immunity* (Singh et al., 2014; Malik et al., 2018; Overacre-Delgoffe et al., 2021). *Nature Medicine* and *Gut* mainly focused on clinical research, while *Cell*, *Science*, and *Immunity* focused on basic experimental research. These prestigious journals have a significant global influence, and are more likely to publish high-quality studies in the future. *Nature Reviews Gastroenterology & Hepatology* and *Nature Reviews Microbiology* had the most influential reviews, indicating that they would be more likely to publish top-level reviews.

These publications are mainly from China and the United States, followed by Italy, Japan, and Germany. China and the United States had the largest Np and stood at the core of global cooperation, which may be due to the high attention and financial support of the two countries in IM and CRC research (Yang S. et al., 2022). Notably, the incidence of CRC is high in China and the United States, and China has surpassed the United States in terms of CRC incidence and mortality (Siegel et al., 2020; Yang et al., 2020a), indicating that China still needs further work in CRC-related research and strengthening international cooperation. The top ten institutions came from China, the United States, and France, demonstrating their good scientific productivity. In China, *Shanghai Jiao Tong University*, *Chinese University of Hong Kong* and *Zhejiang University* published the most articles on IM/CRC. In the United States, *Harvard University*, *Harvard Medical School*, *University of California System*, and *University of North Carolina* made important contributions to IM/CRC research.

Half of the top ten authors were from *Chinese University of Hong Kong* and *Shanghai Jiao Tong University*, which are comprehensive and world-class research universities. The author with most Np and the highest H-index was Yu Jun, an oncologist from *Chinese Univ Hong Kong*, who had made great contributions to the study of IM/CRC, especially on the effect of IM on tumorigenesis of CRC (Nakatsu et al., 2015; Wong et al., 2017b; Yang J. et al., 2022) and the value of IM as a new biomarker in the screening and treatment of CRC (Liang et al., 2017; Wong et al., 2017a; Yu J. et al., 2017; Dai et al., 2018; Liang et al., 2020), and he was at the core of author collaboration in China. In the last few years, she has increasingly focused on the role of specific IM in the treatment of CRC (Li et al., 2021; Sugimura et al., 2021) and the action of the tumor microbiome, enteric virome (Nakatsu et al., 2018) and archaea (Coker et al., 2020) in CRC. Fang Jing-Yuan from *Shanghai Jiao Tong University* has long been interested in the value of IM as a new non-invasive biomarker in the diagnosis of CRC (in close cooperation with Yu Jun) (Liang et al., 2017; Liang et al., 2020) and the role of *F. nucleatum* in CRC (Yu T. et al., 2017; Hong et al., 2021). Garrett Wendy S from *Harvard Med Sch* had the highest TC and published many highly cited papers. His papers focused on the effect of *F. nucleatum* on CRC (Kostic et al., 2013; Mima et al., 2015; Mima et al., 2016), the role of diet (Mehta et al., 2017; Liu et al., 2018) and antibiotics (Cao et al., 2018) in CRC, and found that the human gut bacterial genotoxin colibactin can alkylate DNA to contribute to colorectal carcinogenesis (Wilson et al., 2019).

### 4.2 Research hotspots and frontiers in IM/CRC

Hotspots and frontiers are determined by cluster analysis of common keywords, highly cited papers, and high-IF papers. This study found that the current hot topics of IM/CRC research were concentrated in five perspectives: (1) the effect of IM on tumorigenesis of CRC; (2) the role of IM in the screening of CRC; (3) the effect of IM on CRC treatment; (4) the possible mechanisms of IM involved in CRC; (5) modulating IM for CRC management. Moreover, emerging research, such as chemotherapy, immunotherapy, *Fusobacterium nucleatum*, short-chain fatty acids (SCFAs), and biomarkers, are not only the current hotspots but also the focus of the next several years.

#### 4.2.1 The effect of IM on tumorigenesis of CRC

Some studies (Wong et al., 2017b; Li et al., 2019) have shown that gavage of fecal samples from patients with CRC in germ-free and normal mice can promote the progression of intestinal adenoma and carcinogenesis. Germ-free mice colonized with IM from tumor-bearing mice showed increased tumorigenesis (Zackular et al., 2013), whereas laboratory mice transplanted with IM from wild mice showed increased resistance to colorectal tumorigenesis (Rosshart et al., 2017). IM depletion with antibiotics can result in a significant decrease in subcutaneous tumor and liver metastasis burdens in mice (Zackular et al., 2013; Sethi et al., 2018). Many studies (Konstantinov et al., 2013; Louis et al., 2014; Wong and Yu, 2019; Cheng et al., 2020; Janney et al., 2020) have shown that *Fusobacterium nucleatum*, *Escherichia coli*, *Enterococcus faecalis*, and enterotoxigenic *Bacteroides fragilis* are closely related to CRC tumorigenesis, whereas butyrate-producing bacteria such as *Faecalibacterium*, *Roseburia*, *Clostridium* and *Lachnospiraceae* may inhibit the onset and development of CRC.

##### 4.2.1.1 Fusobacterium nucleatum (F. nucleatum)

Several related studies (Castellarin et al., 2012; Hashemi Goradel et al., 2019; Sánchez-Alcoholado et al., 2020) have shown that *F. nucleatum* infection is prevalent in CRC and is one of the most widely known strains associated with CRC. *F. nucleatum* can promote the adhesion of CRC cells to endothelial cells, extravasation and metastasis (Zhang et al., 2022). The amount of *F. nucleatum* in CRC tissues is negatively correlated with the density of CD3^+^ T cells, and it can promote tumor development by downregulating T cell-mediated adaptive immunity (Mima et al., 2015; Mima et al., 2016). *F. nucleatum* can enhance intestinal tumorigenesis through the TLR4/PAK1 cascade (Wu et al., 2018) and promote glycolysis and tumorigenesis by targeting lncRNA ENO1-IT1 (Hong et al., 2021). Moreover, *F. nucleatum* can enhance intestinal tumorigenesis by modulating the tumor immune microenvironment (Kostic et al., 2013) and promoting chemoresistance in CRC by regulating autophagy (Yu T. et al., 2017). The persistence of *F. nucleatum* in post-neoadjuvant chemoradiotherapy is related to the high recurrence rate of locally advanced rectal cancer, which may be related to the inhibition of immune cytotoxicity (Serna et al., 2020).

##### 4.2.1.2 Escherichia coli (E. coli))

Pathogenic *E. coli* may be a cofactor in the pathogenesis of CRC (Bonnet et al., 2014). Mucosa-associated *pks^+^ E. coli* was found in a significantly high percentage of patients with CRC (Arthur et al., 2012). Colibactin-associated *E. coli* is ubiquitous in the colon mucosa of patients with CRC, and promotes CRC in CRC-susceptible mice (Veziant et al., 2021). The genotoxin colibactin can promote colon tumor growth by modifying the tumor microenvironment (Dalmasso et al., 2014). The toxin released by genotoxic *E. coli* can cause a unique mode of DNA damage to intestinal lining cells, which shows a direct relationship between intestinal bacterial toxins and genetic changes driving CRC development (Pleguezuelos-Manzano et al., 2020). An article in *Science* studied the damage mechanism of colibactin to DNA in human living cells, showing the gut bacterial genotoxin colibactin can alkylate DNA, and the DNA adduct produced by *pks^+^ E. coli* strengthens the support for the participation of colistin in the development or progression of cancer (Wilson et al., 2019).

##### 4.2.1.3 Enterococcus faecalis (E. faecalis)

*E. faecalis* is an opportunistic pathogen in the gut, which is related to a series of hospital infections that are difficult to treat and is also known to be associated with CRC. Its resistance to a series of antibiotics and ability to form biofilms can increase its virulence. *E. faecalis* is also a human intestinal symbiont that produces extracellular superoxide and promotes chromosome instability through the bystander effect induced by macrophages (Wang X. et al., 2012). The abundance of *E. faecalis* in CRC patients is significantly higher than that in healthy individuals (Wang T. et al., 2012). In addition, *in vitro* and *in vivo* studies have shown that *E. faecalis* can produce hydroxyl free radicals, leading to chromosome instability and CRC risk, and can promote the migratory and invasive phenotype of colon cancer cells (Williamson et al., 2022).

##### 4.2.1.4 Enterotoxigenic *Bacteroides fragilis*

ETBF is a bacterium that can produce *Bacteroides fragilis* toxin (BFT), and research shows that colitis driven by ETBF can promote colon carcinogenesis (Sears and Pardoll, 2011; Sears et al., 2014). BFT destroys the colonic epithelial barrier by inducing the cleavage of E-cadherin (a structural protein that inhibits colorectal tumorigenesis) and initiates the cell signal transduction reaction characterized by inflammation and c-Myc-dependent oncogenic hyperproliferation (Wu et al., 2009; Sears and Pardoll, 2011). Significantly, this strain can promote colon tumorigenesis by increasing signal transducer and activator of transcription 3 (STAT3) and T helper type 17 (Th17) response (Wu et al., 2009). A previous study showed that the regulatory response of T cells in the colonization of ETBF triggered IL-17 dependent colon carcinogenesis (Geis et al., 2015). The lncRNA BFAL1 can mediate ETBF-related carcinogenesis in CRC *via* the RHEB/mTOR pathway (Bao et al., 2019).

##### 4.2.1.5 Butyrate-producing bacteria

Butyrate has a series of significant colon health and anti-tumor properties, and can inhibit inflammation and tumorigenesis by regulating immunity, epigenetics, and gene expression (O'Keefe, 2016). Butyrate can inhibit proliferation-promoting miR-92a by reducing miR-17-92a cluster transcription in colon cancer cells, thereby reducing colon cancer cell proliferation and stimulating apoptosis (Hu et al., 2015). Some studies (Wang T. et al., 2012; Weir et al., 2013; Wu et al., 2013) have shown that a significant reduction in butyrate-producing bacteria and an increase in opportunistic pathogens may constitute the main IM imbalance in patients with CRC. Activation of Gpr109a, a receptor for niacin and commercial metallic butyrate, can suppress colonic inflammation and tumorigenesis (Singh et al., 2014). In addition, *Clostridium butyricum* (a butyrate-producing probiotic) can inhibit intestinal tumor progression by regulating Wnt signaling and IM (Chen et al., 2020).

#### 4.2.2 The role of IM in screening of CRC

General risk population screening can reduce the morbidity and mortality associated with CRC. Accurate, noninvasive screening tests can significantly reduce the global health burden of CRC. Multiple studies (Chen et al., 2012; Wang T. et al., 2012; Ahn et al., 2013; Weir et al., 2013; Wu et al., 2013; Zeller et al., 2014; Flemer et al., 2017) have shown that the IM of patients with CRC was different from that of patients without CRC. IM can be used as a novel biomarker for the non-invasive diagnosis of CRC (Zackular et al., 2014; Liang et al., 2017; Wong and Yu, 2019), and metagenomic analysis of IM provides a rich source for CRC screening.

In 2017, Yu et al. (2017). found that 20 gene markers were differentially expressed in CRC and control samples, among which butyryl-coenzyme A dehydrogenase from *F. nucleatum* and RNA polymerase subunit from Micromonas β showed good diagnostic value, with an area under curve (AUC) of 0.84. In 2018, Dai et al. (2018). analyzed metagenomic data from patients with CRC and identified seven species, including *Bacteroides fragilis* and *F. nucleatum* enriched in CRC as potential diagnostic markers that could be used in different populations to distinguish CRC patients from healthy controls (AUC = 0.80). In 2019, *Nature Medicine* published three articles in succession (Thomas et al., 2019; Wirbel et al., 2019; Yachida et al., 2019). Yachida et al. (2019) found that the abundance of *Firmicutes*, *Fusobacteria* and *Bacteroidetes* showed an upward trend with CRC progression; propionate and butyrate were the most abundant metabolites, and the model combining bacterial species, KO genes, and metabolites was the best in terms of resolution, and found a panel of 55 bacterial markers linked to CRC. Wirbel et al. (2019) carried out a meta-analysis of eight shotgun metagenomic studies of CRC and found that the abundance of 29 strains increased in patients with CRC and revealed microbial characteristics specific to CRC. Thomas et al. (2019) conducted a fecal metagenomic meta-analysis from five available datasets and two new cohorts to identify common IM characteristics across different populations of CRC, constructed a CRC disease prediction model containing 16 species (AUC > 0.8), validated it in two additional cohort datasets, and found that the choline trimethylamine lyase gene among the flora genes was enriched in CRC. In 2020, Yang et al. (2020). identified 22 microbial marker genes closely related to CRC and verified these using qPCR. Among them, the biomarker of the gene from *Coprobacillus* showed a high diagnostic value (AUC = 0.93).

In addition, Zeller et al. (2014) showed that combining metagenomic analysis with FOBT could increase the sensitivity of CRC detection. *F. nucleatum* can be used as a biomarker for early CRC screening and prognosis. Wong et al. (2017a) identified *F. nucleatum* as a valuable marker for improving the diagnostic performance of fecal immunochemical tests, with a complementary role in the detection of lesions. Guo et al. (2018) showed that the ratio of *F. nucleatum* to the probiotics *Bifidobacterium* and *Lactobacillus* is a valuable biomarker for early CRC screening. Notably, the characteristic detection of enteric viruses (Nakatsu et al., 2018) and the fungal microbiota (Coker et al., 2019) can also be used for CRC screening.

#### 4.2.3 The effect of IM on treatment of CRC

Efficacy is the most critical factor in the evaluation of antitumor treatment. Research on the impact of IM on cancer therapy is the most important area of cancer microbiome research. It has been confirmed that IM can mediate treatment outcomes of CRC (Wong and Yu, 2019).

##### 4.2.3.1 Chemotherapy

Microorganisms can enhance or decrease the effects of fluoropyrimidines by metabolic interconversion involving bacterial vitamins B_6_ and B_9_ and ribonucleotide metabolism (Scott et al., 2017). IM can control the efficacy of chemotherapy in CRC and immunogenic ileal cell apoptosis can contribute to the prognosis of chemotherapy-treated colon cancer (Roberti et al., 2020). IM dysbiosis can affect the efficacy of 5-fluorouracil (5-FU) in the treatment of CRC (Yuan et al., 2018). Furthermore, Yu et al (Yu T. et al., 2017). found that *F. nucleatum* can promote chemoresistance of CRC, which was related to targeting TLR4 and MYD88 innate immune signals and specific microRNAs to activate the autophagy pathway. Zhang et al (Zhang S. et al., 2019). showed that *F. nucleatum* promotes chemoresistance to 5-FU by upregulating BIRC3 expression in CRC.

##### 4.2.3.2 Immunotherapy

Most studies (Wong and Yu, 2019; Kim and Lee, 2021; Xing et al., 2022) on the correlation between IM and CRC focused on the effect of IM on cancer immunotherapy. IM may be a promising biomarker for CRC immunotherapy (Temraz et al., 2019). A previous study (Xu et al., 2020) showed that IM may affect glycerophospholipid metabolic pathways, thereby modulating the therapeutic potential of PD-1 antibodies in immunotherapy in MSS-type CRC tumor-bearing mice. Many studies have demonstrated that IM can alter the host response to cancer immunotherapy (Schmitt and Greten, 2021; Cai et al., 2022). Additionally, IM can determine whether a patient will respond to cancer immunotherapy and predict treatment-related effectiveness and unfavorable effects (Oh et al., 2021; Yang S. et al., 2022).

#### 4.2.4 The underlying mechanisms of IM involved in CRC

Concretely speaking, the mechanisms of IM affect CRC involve many factors, such as pathogenic bacteria and their virulence factors, inflammation, bacterial metabolites, immunity, oxidative stress, intestinal barrier disruption, and so on (Gagnière et al., 2016; Cheng et al., 2020; Janney et al., 2020).

Firstly, we have discussed how pathogenic bacteria such as *pks^+^ E. coli*, *E. faecalis* and ETBF can induce DNA damage by inducing inflammation and oxidative stress. IM may induce the onset and development of CRC through two modes: the “Alpha-bugs” model (Sears and Pardoll, 2011) and the “Driver-passenger” model (Tjalsma et al., 2012). (1) The “Alpha-bugs” model believes that IM with unique virulence characteristics (Alpha-bugs bacteria), such as ETBF, can directly lead to intestinal epithelial cells carcinogenesis by secreting toxic proteins such as BFT, and ETBF-driven IM changes can cause an abnormal mucosal immune response and accumulation of cancerous intestinal epithelial cells. (2) The “Driver-passenger” model considers certain intestinal bacteria (such as *E. faecalis*, *E. coli* and ETBF) as drivers to induce DNA damage and promote carcinogenesis, subsequently, the inherent drivers will be replaced by some opportunistic pathogens or even beneficial bacteria-passengers such as *Fusobacterium* and *Streptococcus gallolyticus* that are more suitable for survival in the intestinal tumor microecology.

Secondly, chronic intestinal inflammation is generally regarded as a key factor for the progression of colitis-associated CRC (a subtype of CRC that develops directly from inflammatory bowel disease [IBD]), which is supported by the much higher incidence of CRC in patients with IBD, especially those with ulcerative colitis (Arthur et al., 2012; Louis et al., 2014). Microbial symbionts are the key determinants of gut inflammation. IM imbalance can cause host metabolic and immune changes, inducing chronic inflammation and leading to tumor progression (Gagnière et al., 2016; Cani and Jordan, 2018). Crosstalk between microbiota and bile acid also plays a vital role in gastrointestinal inflammation and carcinogenesis (Jia et al., 2018). In addition, the inflammatory tissue environment is conducive to the disturbance of the IM, which is usually characterized by the massive reproduction of specific bacterial species that can use more abundant nutrients in the inflammatory intestine (Zeng et al., 2017). The most cited original study (Arthur et al., 2012) in this paper showed that colitis can promote tumorigenesis by changing the composition of IM and inducing the expansion of genotoxic microorganisms. In summary, colitis-associated CRC mainly occurs through the inflammation-cancer pathway, in which IM plays an important role (Schmitt and Greten, 2021).

Thirdly, IM can affect CRC by means of metabolites, such as secondary bile acids, trimethylamine-N-oxide (TMAO), hydrogen sulfide (promote inflammation and carcinogenesis), and SCFAs such as propionate and butyrate (inhibit inflammation and cancer) (Konstantinov et al., 2013; Louis et al., 2014). Bile acids are metabolized by enzymes from IM, which play a vital role in intestinal immunity, inflammation, and tumors (Cai et al., 2022), and bile acid-microbiome crosstalk can affect gastrointestinal inflammation and carcinogenesis (Jia et al., 2018). Secondary bile acid production may be increased in CRC patients (Wirbel et al., 2019). The IM-derived metabolite, formate, may also exacerbate CRC progression (Ternes et al., 2022). High plasma TMAO (Bae et al., 2014) and hydrogen sulfide (Carbonero et al., 2012) levels are positively associated with high CRC risk. In contrast, IM can promote the excessive proliferation of MSH_2_-deficient colon epithelial cells by providing carbohydrate-derived metabolites such as butyrate, thereby regulating the host immune system (Belcheva et al., 2014). Butyrate can reduce CRC cell proliferation and stimulate apoptosis (Weir et al., 2013; Singh et al., 2014; Hu et al., 2015). A meta-analysis showed that lower fecal concentrations of three major SCFAs (acetic acid, propionic acid, and butyric acid) were associated with a higher risk of CRC (Alvandi et al., 2022).

Last, the immune system mediates the effect of IM on CRC. Changes in crosstalk between the mucosal immune system and IM are considered to be the core defects leading to chronic gut inflammation and cancer progression (Liu et al., 2013). IM can promote tumor growth in mice by regulating immune responses, such as increasing interferon gamma (IFN-γ)-producing T cells and decreasing interleukin 17a (IL-17a)- and IL-10-producing T cells (Sethi et al., 2018). In addition, IM plays a role in memory T cell formation (Liu et al., 2020) and can stimulate CRC cells to produce chemokines that facilitate the recruitment of beneficial T cells to the tumor tissue (Cremonesi et al., 2018). Crosstalk between IM and monocyte-like macrophages can mediate an inflammatory response to promote colitis-related tumorigenesis (Yang et al., 2020b). IM can regulate the host immune system by regulating L-tryptophan metabolism, which plays a crucial role in the balance between intestinal immune tolerance and IM maintenance (Gao et al., 2018). Furthermore, changes in IM can lead to changes in glycerophospholipid metabolism, thereby affecting the therapeutic effect of immunotherapy (Xu et al., 2020).

#### 4.2.5 Regulating IM for prevention and treatment of CRC

IM modification in CRC management is of great significance, as it not only prevents the formation and progression of CRC but also improves the clinical efficacy of cancer patients and reduces adverse events (Fong et al., 2020; Kaźmierczak-Siedlecka et al., 2020). Currently, IM intervention to adjust CRC mainly includes the following aspects:

##### 4.2.5.1 Probiotics and prebiotics

Probiotic supplementation can alter the microbiota structure, modulate inflammatory responses, and prevent CRC. Prebiotic-induced anti-tumor immunity attenuates CRC growth (Li et al., 2020). Specifically, *Bifidobacterium fragilis* may effectively improve chronic inflammation-induced intestinal epithelial damage and prevent the progression of colon tumors (Shao et al., 2021). *Clostridium butyricum* (a butyrate-producing probiotic) inhibits the development of CRC by regulating Wnt signal transduction and IM (Chen et al., 2020). *Lactobacillus casei BL23* may prevent colitis-associated CRC. *Lactobacillus paracasei*-derived extracellular vesicles may reduce intestinal inflammation by enhancing the endoplasmic reticulum stress pathway (Choi et al., 2020). Reuterin, produced by *Lactobacillus reuteri*, can inhibit the growth of CRC cells by altering the redox balance (Bell et al., 2022). In addition, probiotic use can enhance the antitumor effect of 5-FU chemotherapy (Genaro et al., 2019) and is linked to favorable clinical outcomes in immunotherapy (Takada et al., 2021).

##### 4.2.5.2 Diet, nutrition and dietary fiber

IM is a key effector between diet and cancer, and dietary adjustment is expected to reduce the incidence of CRC (O'Keefe, 2016; Sánchez-Alcoholado et al., 2020; Song et al., 2020). For instance, compared with fruits and vegetables, the high intake of red meat seems to be related to the growth of bacteria that may lead to a worse intestinal environment (Feng et al., 2015). Carbohydrate residues stimulate the production of metabolites that maintain mucosal health, while protein residues and fat-stimulated bile acids may lead to proinflammatory and carcinogenic metabolites (O'Keefe, 2016). Dietary emulsifier-induced alterations in the microbiome may promote low-grade inflammation and colon carcinogenesis (Viennois et al., 2017), and a high-fat diet can promote colorectal tumorigenesis by modulating IM and metabolites (Yang J. et al., 2022). Furthermore, polyphenol-rich foods can increase the number of butyrate producers and probiotics, thereby alleviating colitis and inhibiting CRC (Zhao and Jiang, 2021). A diet rich in dietary fiber and whole grains was linked to a lower risk of *F. nucleatum*-positive CRC (Mehta et al., 2017). Moreover, dietary fiber can correct the composition of IM, promote the production of SCFAs, inhibit colorectal carcinogenesis (Bishehsari et al., 2018) and enhance anti-PD-1 efficacy (Zhang et al., 2021).

##### 4.2.5.3 Fecal microbiota transplantation

The main benefits of FMT include regulating the efficacy of immunotherapy, improving bile acid metabolism, and restoring intestinal microbial diversity (Kaźmierczak-Siedlecka et al., 2020). An animal study (Chang et al., 2020) showed that FMT can protect CRC from intestinal injury, upregulation of Toll-like receptors, and chemotherapy-induced toxicity. FMT has a protective effect on colitis-associated cancer by restoring IM, reducing proinflammatory factors, increasing anti-inflammatory factors, and inducing regulatory T cells (Wang et al., 2019). Furthermore, FMT can improve the efficacy of cancer immunotherapy and reduce its side effects (Kang and Cai, 2021). For example, some studies demonstrated that FMT can enhance the efficacy of anti-PD-1 immunotherapy (Huang et al., 2022) and effectively treat immunotherapy-associated colitis (Wang et al., 2018).

##### 4.2.5.4 Provision of specific microbiota

*F. nucleatum*-specific phages isolated were linked to dextran nanoparticles loaded with CRC chemotherapeutics to form phage-guided nanomedicines, which could effectively hinder the growth of *F. nucleatum*, prolong the survival of CRC mice, reduce the number of adenomas, and increase the efficacy of chemotherapy in CRC (Zheng et al., 2019). A study (Dong et al., 2020) screened a specific *F. nucleatum*-binding M13 phage to regulate IM and reshape the tumor immune microenvironment for CRC, which prolonged the overall survival of orthotopic CRC mice. In addition, the introduction of *Spirillum hepaticum* into CRC mice increased tumor cytotoxic lymphocyte infiltration and inhibited tumor growth; therefore, the introduction of immunogenic intestinal bacteria can promote T follicular helper cell-related anti-tumor immunity, providing a therapeutic method for CRC (Overacre-Delgoffe et al., 2021).

##### 4.2.5.5 Antibiotics

Increasing evidence has revealed that antibiotic use can change IM and is linked to an increased risk of CRC. Long-term antibiotic use in early middle adulthood is also linked to an increased risk of colorectal adenomas (Cao et al., 2018). A clinical study (Zhang J. et al., 2019) examining the association between oral antibiotic use and CRC risk found that oral antibiotics increased the risk of colon cancer and decreased that of rectal cancer. An animal study (Yuan et al., 2018) showed that antibiotic use destroyed the IM of mice, resulting in a reduction in the antitumor efficacy of 5-FU. In addition, antibiotic use has been linked to worse clinical outcomes in immunotherapy-treated patients with cancer (Chen et al., 2021; Tsikala-Vafea et al., 2021).

##### 4.2.5.6 Traditional Chinese medicine

Traditional Chinese medicine may manage CRC by adjusting IM. For example, neohesperidin can prevent colorectal tumorigenesis by altering IM (Gong et al., 2019). Ophiocordyceps sinensis can attenuate colitis-associated cancer by increasing the abundance of probiotics (Ji et al., 2021). YYFZBJS inhibits CRC progression by reforming IM and inhibiting regulatory T cell generation (Sui et al., 2020). Gegen Qinlian Decoction can enhance PD-1 immunotherapy in CRC by remodeling microsatellites to stabilize the IM and tumor microenvironment (Lv et al., 2019).

### 4.3 Limitations of the research

Our study has some limitations. First, only papers in the SCI-E of WoSCC were searched and included; this could not cover all studies in multiple databases worldwide, which may cause some incompleteness in the results. Second, bibliometric tools cannot currently analyze the entire content of papers, and some concrete information may be ignored. The analysis of high-cited papers and high-IF papers made up for these shortcomings and limitations. Third, this study only analyzed papers at the current stage, and some newly published papers may have higher significance but are cited less currently.

## 5 Conclusions

In the past ten years, interest in IM/CRC research has increased rapidly, and researchers from China and the United States have made important contributions to this field. We found that IM not only affects the onset and development of CRC, but may also be used as a biomarker to screen CRC patients, predict the prognosis of CRC, and determine the efficacy of cancer treatment. Determining the dynamics of IM may help to elucidate the pathogenesis of CRC. Fecal detection of microbial markers based on metagenomics can effectively quantify IM, and is expected to become a new method for early CRC screening. Given the regional variation, it is necessary to build localized baseline and disease models to predict the risk of CRC. Modifying IM can not only prevent CRC but also improve the clinical efficacy of cancer treatment. IM-centric interventions may be the next breakthrough for the prevention, screening and treatment of CRC. We can change the IM of CRC patients by diet, probiotics and FMT, and host’ response to CRC treatment. Knowing the mechanism of the links between IM and CRC, and then adjusting IM to prevent and treat CRC, is a captivating direction for research. With the sustained development of IM/CRC research, using IM as a screening, prognostic, and predictive biomarker will be extremely likely in the future. In short, this study showed the global research status of IM/CRC, offers scholars a better understanding of the development trend of IM/CRC, and indicates an overall perspective for further in-depth study.

## Data availability statement

The original contributions presented in the study are included in the article/**Supplementary Material**. Further inquiries can be directed to the corresponding authors.

## Author contributions

SY: manuscript writing, data collection and inspection, investigation, and figure preparation. SH: manuscript revision, data collection and inspection, and figure preparation. XZ: manuscript check and review, methodology, and supervision. HY: manuscript review and polishing, methodology, and supervision. All authors contributed to the article and approved the submitted version.
