# Supplementary material for: Global research on the crosstalk between intestinal microbiome and colorectal cancer: A visualization analysis
Source: Front Cell Infect Microbiol. 2023 Mar 15;13:1083987. doi: 10.3389/fcimb.2023.1083987 (PMC10050574; doi:10.3389/fcimb.2023.1083987)
Supplement: Supplementary file 1 [file Table_1.docx]

# Searches:

1: TI OR AB OR AK= (“Colorectal Neoplasm*” OR “Colorectal Tumor*” OR “Colorectal Cancer*” OR “Colorectal Carcinoma*” OR “Rectal Neoplasm*” OR “Rectum Neoplasm*” OR “Rectal Tumor*” OR “Rectum Tumor*” OR “Rectal Cancer*” OR “Rectum Cancer*” OR “Cancer of Rectum” OR “Cancer of the Rectum” OR “Colonic Neoplasm*” OR “Colon Neoplasm*” OR “Colonic Tumor*” OR “Colon Tumor*” OR “Colonic Cancer*” OR “Colon Cancer*” OR “Colonic Adenocarcinoma*” OR “Colon Adenocarcinoma*” OR “Cancer of Colon” OR “Cancer of the Colon”)

2: TI OR AB OR AK = (“Gut Microb*” OR “Gut Microflora” OR “Gut Flora” OR “Gut Microbial Flora” OR "Gut Microecology" OR “Gut Mucosal Microb*” OR “Gut Mucosal Microflora” OR “Gut Mucosal Flora” OR “Gut Mucosal Microbial Flora” OR "Gut Mucosal Microecology" OR “Intestinal Microb*” OR “Intestinal Microflora” OR “Intestinal Flora” OR “Intestinal Microbial Flora” OR "Intestinal Microecology" OR “Intestinal Mucosal Microb*” OR “Intestinal Mucosal Microflora” OR “Intestinal Mucosal Flora” OR “Intestinal Mucosal Microbial Flora” OR "Intestinal Mucosal Microecology" OR “Enteric Microb*” OR “Enteric Microb*” OR “Enteric Microflora” OR “Enteric Flora” OR “Enteric Microbial Flora” OR " Enteric Microecology" OR “Gastrointestinal Microb*” OR “Gastrointestinal Microflora” OR “Gastrointestinal Flora” OR “Gastrointestinal Microbial Flora” OR “Gastrointestinal Microbial Communit*” OR "Gastrointestinal Microecology" OR “Fecal Microb*” OR “Fecal Microflora” OR “Fecal Flora” OR “Fecal Microbial Flora” OR “Faecal Microb*” OR “Faecal Microflora” OR “Faecal Flora” OR “Faecal Microbial Flora” OR “Stool Microb*” OR “Stool Microflora” OR “Stool Flora” OR “Stool Microbial Flora” OR “Gut Bacteri*” OR “Intestinal Bacteri*” OR “Gastrointestinal Bacteri*” OR “Fecal Bacteri*” OR “Faecal Bacteri*” OR “Stool Bacteri*” OR “Enteric Bacteri*”)

3: #1 AND #2 and Science Citation Index Expanded (SCI-EXPANDED) (Web of Science Index) and 2022 (Exclude – Publication Years) and Meeting Abstract or Editorial Material or Proceeding Paper or Book Chapters or Correction or News Item or Letter or Early Access or Retracted Publication or Data Paper (Exclude – Document Types) and 2011 or 2010 or 2009 or 2008 or 2007 or 2006 or 2005 or 2004 (Exclude – Publication Years)

Query link:

https://www.webofscience.com/wos/woscc/summary/9e7ce106-23a6-42e6-b81e-12d2f248e186-56831e3c/times-cited-descending/1
