# Supplementary material for: Global research on the crosstalk between intestinal microbiome and colorectal cancer: A visualization analysis
Source: Front Cell Infect Microbiol. 2023 Mar 15;13:1083987. doi: 10.3389/fcimb.2023.1083987 (PMC10050574; doi:10.3389/fcimb.2023.1083987)
Supplement: Supplementary file 2 [file Table_2.docx]

**Additional TABLE 1.** The high-impact factor(IF＞40) original research in GM/CRC.

| **Rank** | **Title** | **DOI** | **Year** | **Journals** | **IF** | **JCR** | **TC** |
| --- | --- | --- | --- | --- | --- | --- | --- |
| 1 | Intestinal Inflammation Targets Cancer-Inducing Activity of the Microbiota | 10.1126/science.1224820 | 2012 | Science | 63.714 | Q1 | 1280 |
| 2 | Activation of Gpr109a, Receptor for Niacin and the Commensal Metabolite Butyrate, Suppresses Colonic Inflammation and Carcinogenesis | 10.1016/j.immuni.2013.12.007 | 2014 | Immunity | 43.474 | Q1 | 1126 |
| 3 | Fusobacterium nucleatum Promotes Chemoresistance to Colorectal Cancer by Modulating Autophagy | 10.1016/j.cell.2017.07.008 | 2017 | Cell | 66.850 | Q1 | 783 |
| 4 | Two FOXP3(+)CD4(+) T cell subpopulations distinctly control the prognosis of colorectal cancers | 10.1038/nm.4086 | 2016 | Nat. Med. | 87.241 | Q1 | 446 |
| 5 | Patients with familial adenomatous polyposis harbor colonic biofilms containing tumorigenic bacteria | 10.1126/science.aah3648 | 2018 | Science | 63.714 | Q1 | 435 |
| 6 | Wild Mouse Gut Microbiota Promotes Host Fitness and Improves Disease Resistance | 10.1016/j.cell.2017.09.016 | 2017 | Cell | 66.850 | Q1 | 357 |
| 7 | Metagenomic and metabolomic analyses reveal distinct stage-specific phenotypes of the gut microbiota in colorectal cancer | 10.1038/s41591-019-0458-7 | 2019 | Nat. Med. | 87.241 | Q1 | 346 |
| 8 | Meta-analysis of fecal metagenomes reveals global microbial signatures that are specific for colorectal cancer | 10.1038/s41591-019-0406-6 | 2019 | Nat. Med. | 87.241 | Q1 | 338 |
| 9 | Mutational signature in colorectal cancer caused by genotoxic pks(+)E. coli | 10.1038/s41586-020-2080-8 | 2020 | Nature | 69.504 | Q1 | 297 |
| 10 | Metagenomic analysis of colorectal cancer datasets identifies cross-cohort microbial diagnostic signatures and a link with choline degradation | 10.1038/s41591-019-0405-7 | 2019 | Nat. Med. | 87.241 | Q1 | 276 |
| 11 | Gut Microbial Metabolism Drives Transformation of Msh2-Deficient Colon Epithelial Cells | 10.1016/j.cell.2014.04.051 | 2014 | Cell | 66.850 | Q1 | 267 |
| 12 | The human gut bacterial genotoxin colibactin alkylates DNA | 10.1126/science.aar7785 | 2019 | Science | 63.714 | Q1 | 219 |
| 13 | Critical Role for the DNA Sensor AIM2 in Stem Cell Proliferation and Cancer | 10.1016/j.cell.2015.06.001 | 2015 | Cell | 66.850 | Q1 | 206 |
| 14 | Host-Microbe Co-metabolism Dictates Cancer Drug Efficacy in C. elegans | 10.1016/j.cell.2017.03.040 | 2017 | Cell | 66.850 | Q1 | 126 |
| 15 | Butyrate inhibits pro-proliferative miR-92a by diminishing c-Myc-induced miR-17-92a cluster transcription in human colon cancer cells | 10.1186/s12943-015-0450-x | 2015 | Mol. Cancer | 41.444 | Q1 | 99 |
| 16 | The itinerary of Streptococcus gallolyticus infection in patients with colonic malignant disease | 10.1016/S1473-3099(13)70107-5 | 2013 | Lancet Infect. Dis. | 71.421 | Q1 | 85 |
| 17 | SYK-CARD9 Signaling Axis Promotes Gut Fungi-Mediated Inflammasome Activation to Restrict Colitis and Colon Cancer | 10.1016/j.immuni.2018.08.024 | 2018 | Immunity | 43.474 | Q1 | 80 |
| 18 | Chemotherapy-induced ileal crypt apoptosis and the ileal microbiome shape immunosurveillance and prognosis of proximal colon cancer | 10.1038/s41591-020-0882-8 | 2020 | Nat. Med. | 87.241 | Q1 | 66 |
| 19 | Fusobacterium nucleatum persistence and risk of recurrence after preoperative treatment in locally advanced rectal cancer | 10.1016/j.annonc.2020.06.003 | 2020 | Ann. Oncol. | 51.769 | Q1 | 34 |
| 20 | Microbiota-specific T follicular helper cells drive tertiary lymphoid structures and anti-tumor immunity against colorectal cancer | 10.1016/j.immuni.2021.11.003 | 2021 | Immunity | 43.474 | Q1 | 12 |

**Additional TABLE 2.** The high-impact factor (IF＞40) reviews related to GM/CRC.

| **Rank** | **Title** | **First author** | **Year** | **Journals** | **IF** | **JCR** | **TC** |
| --- | --- | --- | --- | --- | --- | --- | --- |
| 1 | The gut microbiota, bacterial metabolites and colorectal cancer | Louis, P | 2019 | Nat. Rev. Microbiol. | 78.297 | Q1 | 1372 |
| 2 | Bile acid-microbiota crosstalk in gastrointestinal inflammation and carcinogenesis | Jia, W | 2016 | Nat. Rev. Gastroenterol. Hepatol. | 73.082 | Q1 | 551 |
| 3 | Diet, microorganisms and their metabolites, and colon cancer | O'Keefe, SJD | 2016 | Nat. Rev. Gastroenterol. Hepatol. | 73.082 | Q1 | 479 |
| 4 | A bacterial driver-passenger model for colorectal cancer: beyond the usual suspects | Tjalsma, H | 2018 | Nat. Rev. Microbiol. | 78.297 | Q1 | 473 |
| 5 | Gut microbiota in colorectal cancer: mechanisms of action and clinical applications | Wong, SH | 2017 | Nat. Rev. Gastroenterol. Hepatol. | 73.082 | Q1 | 310 |
| 6 | Contributions of the microbial hydrogen economy to colonic homeostasis | Carbonero, F | 2017 | Nat. Rev. Gastroenterol. Hepatol. | 73.082 | Q1 | 163 |
| 7 | Gut microbiota-mediated inflammation in obesity: a link with gastrointestinal cancer | Cani, PD | 2019 | Nat. Rev. Gastroenterol. Hepatol. | 73.082 | Q1 | 162 |
| 8 | Early-onset colorectal cancer: initial clues and current views | Hofseth, LJ | 2018 | Nat. Rev. Gastroenterol. Hepatol. | 73.082 | Q1 | 115 |
| 9 | Host-microbiota maladaptation in colorectal cancer | Janney, A | 2018 | Nature | 69.504 | Q1 | 104 |
| 10 | Rising incidence of early-onset colorectal cancer - a call to action | Akimoto, N | 2018 | Nat. Rev. Clin. Oncol. | 65.011 | Q1 | 94 |
